# Development and application of an LC–MS/MS method for quantification of fosmidomycin in human and rat plasma

**DOI:** 10.1186/s12936-025-05489-1

**Published:** 2025-07-25

**Authors:** Christoph Pfaffendorf, Keanu D. Sackmann, Johannes Mischlinger, Jean Claude Dejon-Agobé, Oumou Maïga-Ascofaré, Ebenezer Ahenkan, Ayôla Akim Adegnika, Michael Ramharter, Philipp Uhl, Gert Fricker, Sebastian G. Wicha

**Affiliations:** 1 Institute of Pharmacy, Dept. of Clinical Pharmacy, University of Hamburg, Bundesstrasse 45, 20146 Hamburg, Germany; 2 Centre for Tropical Medicine, Department of Medicine, Bernhard Nocht Institute for Tropical Medicine, University Medical Center Hamburg-Eppendorf, Hamburg, Germany; 3 German Centre for Infection Research, Partner Site Hamburg-Lübeck-Borstel-Riems, Hamburg, Germany; 4 Institute of Pharmacy and Molecular Biotechnology, Ruprecht-Karls-University, Heidelberg, Germany; 5 Centre de Recherches Médicales de Lambaréné, Lambaréné, Gabon; 6 Kumasi Centre for Collaborative Research in Tropical Medicine, Kumasi, Ghana; 7 Infectious Disease Epidemiology, Bernhard Nocht Institute for Tropical Medicine, Hamburg, Germany; 8 Department of Implementation Research, Bernhard Nocht Institute for Tropical Medicine, Hamburg, Germany; 9 Institut Für Tropenmedizin, Eberhard-Karls-Universität Tübingen, Tübingen, Germany; 10 German Centre for Infection Research (DZIF), Tübingen, Germany; 11 Department of Pharmacology, Kwame Nkrumah University of Science and Technology, Kumasi, Ghana

**Keywords:** Fosmidomycin, LC–MS/MS, Pharmacokinetics, Bioanalysis, Malaria

## Abstract

**Background:**

Malaria still poses a significant burden on global health, with millions of cases reported annually and rising resistance to current treatments, emphasizing the need for new therapeutic strategies. Fosmidomycin, initially recognized for its antibacterial properties, has emerged as a promising candidate in the fight against malaria.

**Methods:**

In this study, a sensitive and robust LC–MS/MS method for quantifying fosmidomycin in human and rat plasma was developed and validated. Plasma samples were prepared using a simple protein precipitation method with 10% trichloroacetic acid (TCA). The assay featured a rapid run time of 5 min, and validation was performed according to the European Medicines Agency's guidelines.

**Results:**

The method validation confirmed its selectivity, linearity, accuracy, precision, and stability. Notably, the calibration range was established from 0.25 to 15 mg/L, demonstrating improvements over previous methodologies with lower limits of quantification of 0.5–1.0 mg/L. Using the developed LC–MS/MS method, plasma samples were analysed from a clinical trial conducted in Gabon, as well as from a pharmacokinetic study involving male Wistar rats, revealing viable pharmacokinetic profiles for fosmidomycin.

**Conclusions:**

These findings confirm the utility of the developed analytical method for supporting the clinical development of fosmidomycin as a potential therapy for malaria.

**Supplementary Information:**

The online version contains supplementary material available at 10.1186/s12936-025-05489-1.

## Background

Malaria remains a significant global health challenge. Despite advances in treatment and prevention, the disease continues to significantly contribute to global morbidity and mortality, with approximately 247 million cases and an estimated 619,000 deaths reported in 2022 [1]. A particularly concerning development is the emergence and spread of resistance to artemisinin derivatives, the backbone of current first-line antimalarial therapies. Initially detected in Southeast Asia, there is increasing concern about its possible spread to Africa, where the malaria burden is highest [2, 3]. This situation highlights the urgent necessity for innovative therapeutic strategies. As artemisinin resistance and other drug-resistant strains of *Plasmodium* spp. jeopardize the effectiveness of existing treatments, developing new, potent antimalarial interventions is crucial to effectively combat this persistent public health threat and reduce the global malaria burden.

One of these new antimalarials is fosmidomycin. Originally discovered in 1980 within the microbial flora of *Streptomyces lavendulae* [4], it has regained scientific interest due to its effectiveness against *Plasmodium* spp., the parasites responsible for malaria. Initially noted for its efficacy against gram-negative bacteria, its renewed interest in recent years is centered on its potential utility against malaria [5–7]. Recent clinical trials have shown promising results in assessing fosmidomycin for the treatment of uncomplicated malaria [8]. A comprehensive understanding of fosmidomycin’s pharmacokinetics is crucial for optimizing its therapeutic efficacy. This understanding necessitates developing robust and reliable analytical methods to accurately quantify drug concentrations during preclinical and clinical development.

Previously, pharmacokinetic analyses of fosmidomycin mostly employed disk diffusion assays, but these methods, with a lower limit of quantification (LLOQ) of 1 µg/mL, lacked sensitivity and are susceptible to interference from other antibiotics and biological variability [9]. Alternative methods, such as capillary electrophoresis assays with a lower LLOQ of 0.5 µg/mL, have also been introduced, but they require a work intensive sample preparation and show problems of interference with endogenous compounds with fosmidomycin at the used UV wavelength [10]. Furthermore, both methods are not standard practice in most analytical labs for the quantification of small molecules.

In the light of these limitations, a novel, robust, and sensitive liquid chromatography- tandem mass spectrometry (LC–MS/MS) method for quantifying fosmidomycin in human and rat plasma was developed and validated. This analytical approach offers enhanced sensitivity, is less prone to biological variability and uses a more standard technique, addressing the shortcomings of previous methodologies. Hence, this method shall facilitate the preclinical and clinical development of fosmidomycin as a viable antimalarial treatment, demonstrating its applicability and reliability for fosmidomycin analysis in both human patients and rat models.

## Methods

### Chemicals and reagents

Fosmidomycin (FSM) was purchased from Chiracon GmbH (Luckenwalde, Germany). The internal standard (IS) fosfomycin (FOF), water, acetonitrile, trichloroacetic acid (TCA), ammonium formiate, human serum (from male AB plasma) and formic acid were procured from Sigma-Aldrich (Steinheim, Germany) and were LC–MS Grade. Blank human heparin plasma was procured from the patients of the MultiMal Study at the Centre de Recherches Médicales de Lambaréné (CERMEL) (Lambaréné, Gabon) and blank rat plasma was provided from the Institute of Pharmacy and Molecular Biotechnology (IPMB) (Heidelberg, Germany).

### Equipment

The method was developed on a 1290 Infinity high performance liquid chromatography (HPLC) II (Agilent Technologies, California, USA) coupled to a QTRAP 5500 (SCIEX, Framingham, Massachusetts, USA) electrospray ionization (ESI) mass spectrometer. A reverse-phased column (Ascentis® Express 90 Å AQ-C18 column (150 × 3 mm, 5 µm particle size) from Supelco (Bellafonte, USA) was used.

### Standard solutions

#### Analyte stock

A 1 mg/mL stock of FSM was produced in deionized water. Two separate stocks were made for calibration standards and quality controls. The stocks were stored at − 80 °C until the day of the analysis. Using the 1 mg/mL stock, spiking solutions for calibration standards and quality controls were produced. These spiking solutions were freshly prepared on the day of sample preparation and not stored.

#### Internal standard solution

A 1 mg/mL stock of FOF in deionized water was prepared and diluted to 4 µg/mL with a 10 mM ammonium formate buffer containing 0.25% formic acid.

#### Standard samples

Using the spiking solutions, calibration standards and quality controls were prepared. Each sample was diluted 1:20 with plasma. Calibration standards and quality controls were stored up to 2 weeks at − 80 °C. On the day of analysis, the samples were thawed at room temperature and immediately used.

#### Sample preparation

20 µL of the clinical sample, calibration standard or quality control were mixed with 80 µL of the internal standard solution and vortexed for 5 s. An aliquot of 50 µL of a 10% TCA in water solution was added to the mixture and vortexed for 10 s. The samples were then centrifuged at 4 °C for 20 min at, 17,968 *g*. 80 µL of the supernatant was transferred to a HPLC vial and stored at 4 °C until the start of the measurement.

#### Liquid chromatography-tandem mass spectrometry conditions

Different gradient elution programs were assessed to reach a sufficient retention. Finally, a 100% aqueous isocratic method was found to perform best. The eluent used in was a solution of 10 mM ammonium formate with 0.1% formic acid. The run time for the analysis was set at 5 min, with a flow rate of 0.3 mL/min and a column temperature maintained at 40 °C. An injection volume of 10 µL was employed. The retention time for FSM was 2.32 min, while the retention time for FOF was recorded at 2.24 min. The samples were stored at 4 °C in the autosampler during a run.

Direct injection using negative mode was used to find the optimal parent ion (Q1) and fragments (Q3) for FSM and FOF. The fragment with the strongest signal was chosen as the quantifier for FSM, which was m/z 181.9–135.9 (Q1-Q3). Additionally, the fragment with the second-highest signal was used as the quantifier, which was m/z 181.9–78.8 (Q1-Q3). The fragment chosen for FOF was m/z 136.9–78.9 (Q1-Q3). Flow injection analysis (FIA) was used to optimize the source parameters. The final source parameters and parameters used for each analyte are found in the supplement (S1, S2).

### Bioanalytical method validation

The method was validated based on the European Medicines Agencies (EMA) Guideline for bioanalytical method validation [11]. All validation experiments were performed using human plasma samples. To cross validate the method for rat plasma, the tests for selectivity, and one accuracy and precision run were additionally performed with rat plasma.

#### Selectivity and carry over

To evaluate the selectivity of the method, blank plasma samples were analyzed both with the internal standard (blank) and without it (double blank). These were then compared to plasma samples spiked with FSM at the lowest calibration level. For the method to be considered selective, the samples should exhibit a signal that is less than 20% of the lowest calibrator and less than 5% of the internal standard's signal. To assess carry over, a solvent sample was injected following the highest calibrator, and its signal was compared to that of the lowest calibrator.

#### Linearity and lower limit of quantification

The linearity of the calibration curves, spanning concentrations from 0.25 to 15 mg/L, was assessed over three consecutive days. On each day, six concentration levels were evaluated in duplicate. Calibration curves were created by plotting the area ratios (analyte to internal standard) against the nominal concentrations, employing a linear least squares regression model weighted by 1/x^2^. The limit of quantification was defined as the lowest concentration on the calibration curve that can still be measured with acceptable accuracy and precision.

#### Accuracy and precision

Accuracy and precision were evaluated over three consecutive days. Four levels of quality control samples were measured in sets of five. Both interday and intraday accuracy and precision were assessed. To be considered accurate, the sample concentrations should not deviate more than ± 15% from the nominal concentration, except for the lower limit of quantification (LLOQ), which should be within ± 20%. Precision is acceptable if the coefficient of variation (CV) is no greater than 15%, or 20% for the LLOQ.

#### Matrix and anticoagulant effect

The matrix effect was evaluated by spiking blank plasma samples from six individuals with FSM after sample preparation and comparing their peak areas, normalized by the internal standard, to those obtained without the plasma matrix. 2 samples were prepared for each individual at the concentration level of QCL and QCH. Additionally, the influence of the anticoagulant heparin was examined by comparing these peak areas to those from samples prepared using human serum. For each sample, the peak area ratios were calculated, and the coefficient of variation (%CV) was used to assess variability. To ensure minimal interference from the matrix, the variability should not exceed 15% CV.

#### Stability

For the stability tests, five replicates each of low, medium, and high-quality control levels were used, except for autosampler stability, which evaluated only low and high concentration levels. In the bench top stability test, samples were kept at room temperature (23 °C) for 90 min before analysis. For refrigerator stability, samples were stored at 4 °C for 6 h before being analyzed. The freeze–thaw stability test involved storing samples at − 80 °C, then thawing and refreezing them for at least 12 h, repeating this cycle three times, with analyses conducted immediately after the third thaw. For autosampler stability, samples were maintained in the autosampler at 4 °C and analysed after 8, 10, and 12 h.

#### Accelerated stability study

To estimate the long-term stability of FSM in plasma at − 20 °C and − 80 °C, an accelerated stability study was conducted using the Arrhenius equation (Eq. 1) as a model to understand the temperature dependence of degradation. The Arrhenius equation provides an approximation of how a chemical reaction's rate changes with temperature, based on the concept that activation energy must be overcome for the reaction to proceed.

1 $$k_{e} = A \cdot {\text{ exp}}( - E_{A} /R \cdot T)$$

*k*_*e*_: Reaction rate, *A*: Arrhenius factor,* E*_*A*_ activation energy [J∙mol^−1^], *R*: Universal gas constant [8.314 J∙mol^−1^ ∙ K^−1^], *T*: Temperature [K].

In the context of a stability study, *kₑ* represents the rate of degradation. By taking the natural logarithm of Eqs. 1, 2 is obtained, which describes the Arrhenius plot, where ln(*kₑ*) is plotted against *1/T*. The slope of this function is given by *E*_*A*_*/R*, and the y-intercept is ln(*A*).

2 $${\text{ln}}\left( {{\text{k}}_{e} } \right) \, = \, - E_{A} /R \cdot {1}/T + {\text{ ln}}\left( A \right)$$

The slope and intercept of the function can be experimentally estimated by measuring *kₑ* at different temperatures and applying linear regression. To determine *kₑ*, FSM in plasma was stored at various temperatures for different durations. The exact conditions and times are shown in the supplement (S3). Both low and high-quality control levels (LQC and HQC) were stored and measured in triplicate. The degradation of FSM was assumed to follow first-order kinetics, as described by Eq. 3. By plotting ln(C(t)) against time and fitting a linear regression, the slope provides *kₑ,* as illustrated in Eq. 4.

3 $$C\left( t \right) \, = C_{0} \cdot {\text{exp}}\left( { - k_{e} \cdot t} \right)$$

4 $${\text{ln}}\left( {C\left( t \right)} \right) \, = {\text{ ln}}\left( {C_{0} } \right) - k_{e} \cdot t$$

*C*: concentration (mg/L), *C*_*0*_: initial concentration [mg/L], *k*_*e*_: degradation rate, *t*: time [h].

### Pharmacokinetic application

#### Pharmacokinetic analysis

In this study, the `PKNCA` package (version 0.10.2) [12] was employed for the non-compartmental analysis of pharmacokinetic data, utilizing the programming language R (version 4.3.2) [13].

#### Pharmacokinetic application in humans

The assay was developed as part of the MultiMal clinical trial, a phase II proof of concept trial testing novel combination therapies against uncomplicated plasmodium falciparum infections. The study arm containing FSM was conducted at CERMEL in Lambaréné Gabon. Plasma samples were taken at 0, 0.25, 0.5, 0.75, 1.5, 3, 5, 8, 24, 48, and 168 h. Blood samples were collected in 2 mL Li-heparin tubes and subsequently centrifuged at 1300 *g* for 10 min at room temperature to separate the plasma. The resulting plasma was carefully transferred into polypropylene tubes. To preserve sample integrity, the tubes were then stored at − 80 °C until the time of analysis. The samples were transported from Lambaréné to Hamburg for the LC–MS analysis. The transport was done on dry ice with constant temperature tracing. The clinical trial was approved by the relevant Independent Ethics Committees, national Institutional Review Boards, and local regulatory authorities. The study protocol was registered online before start of recruitment into this clinical trial (pactr.samrc.ac.za, PACTR202008909968293). All patients taking part in the study gave informed consent. In this application, 5 adult patients (4 female/male) which had rich data which allowed for non-compartmental analysis were included.

#### Pharmacokinetic application in rats

In cooperation with the IPBM in Heidelberg, Germany, a second pharmacokinetic study was performed in male Wistar rats. All animal studies were approved by the Animal Care and Use committees from Regierungspräsidium Karlsruhe (Karlsruhe, Germany; reference number 35-9185.81/G-223/21).

The rats received 5 mg FSM dissolved in 0.9% NaCl p.o. by gavage. Plasma samples were taken at 0,0.5,1,1.5,3,6, and 8 h. Blood samples were collected using monovettes at the eye of the rats into Li-heparin tubes. The blood samples were centrifuged at 10,956 g for 3 min to separate the plasma. The plasma was transferred into a polypropylene tube and stored at − 80 °C. The samples were shipped from Heidelberg to Hamburg for the LC–MS analysis. The shipping was done in dry ice.

## Results

### Bioanalytical method validation

The method was successfully validated for the use with human plasma. Furthermore, the cross validation for rat plasma was also successful. The results of the validation are presented below.

#### Selectivity and carry-over

**Fig. 1:**
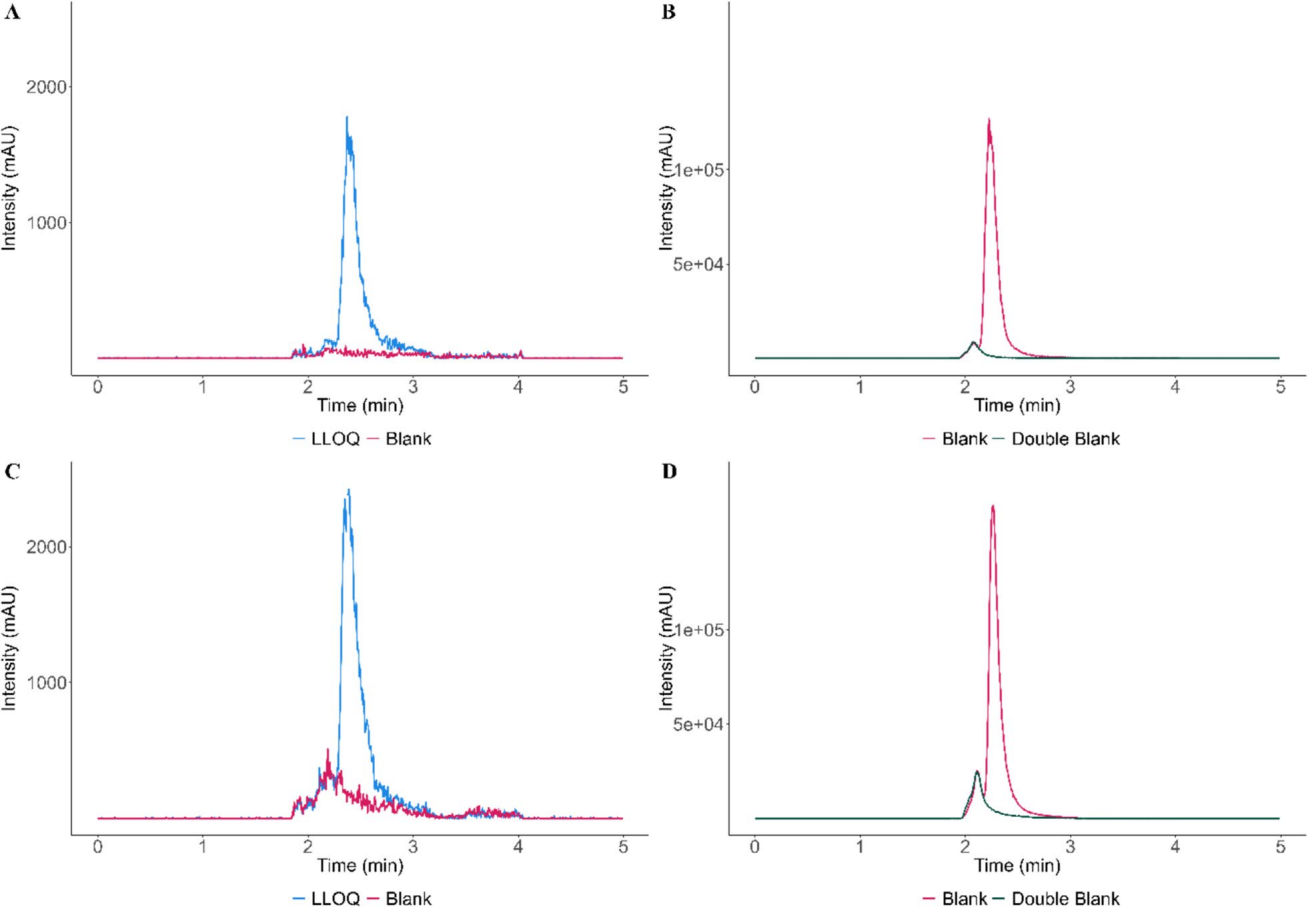
**A** Representative chromatograms of fosmidomycin comparing a LOQ sample in human plasma to a blank human plasma sample. **B** Representative chromatograms of the IS comparing a blank human plasma sample to a double blank human plasma sample. **C** Representative chromatograms of fosmidomycin comparing a LOQ sample in rat plasma to a blank rat plasma sample. **D** Representative chromatograms of the IS comparing a blank rat plasma sample to a double blank rat plasma sample

**Fig. 2:**
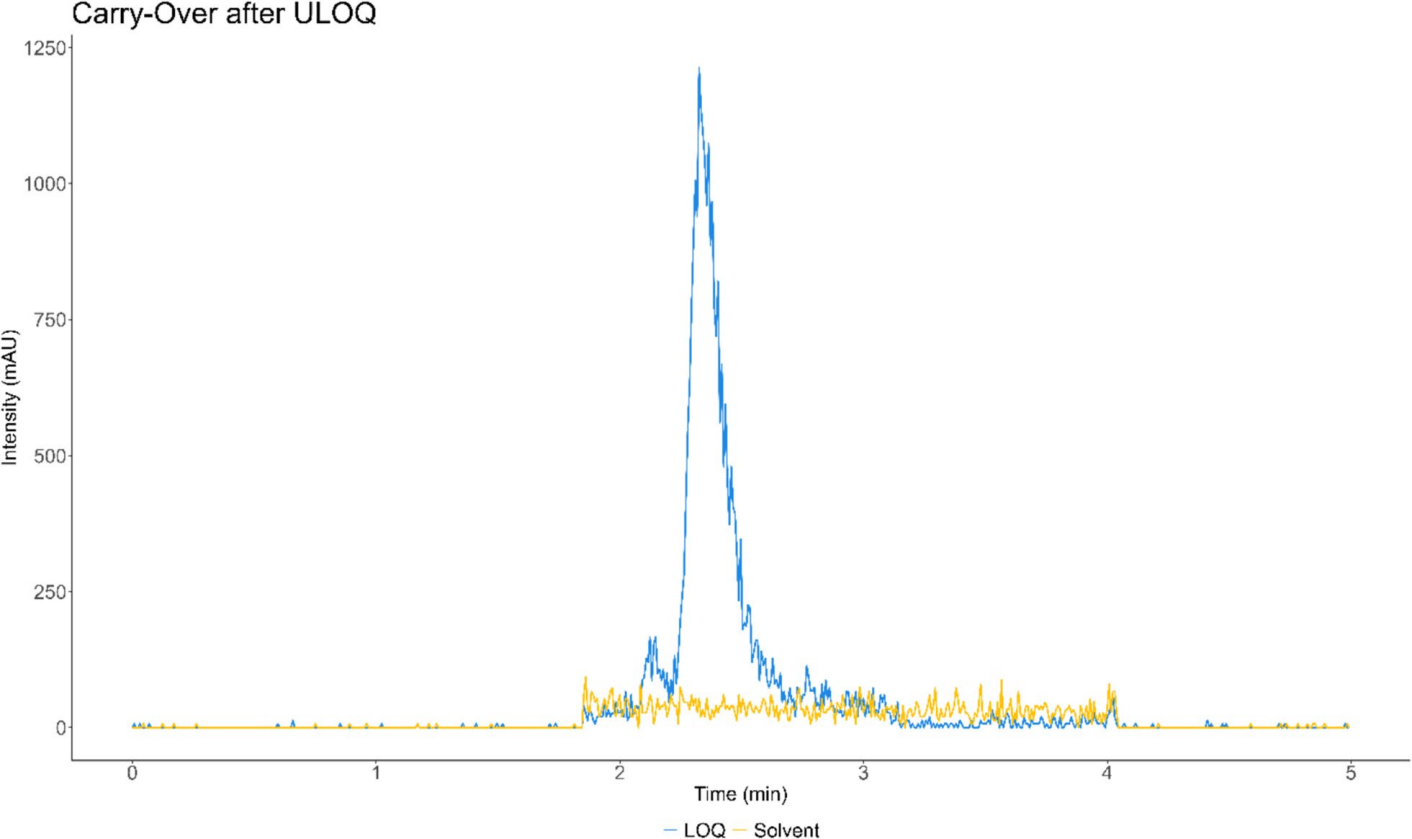
Fosmidomycin signal of a solvent injection after an injection of the upper limit of quantification (ULOQ) compared to the signal of the LLOQ

Selectivity was successfully proven for human and rat plasma, as the peaks observed in the blank human/rat plasma samples were less than 20% of the area observed in the LLOQ sample. The chromatogram of the IS showed a peak in the double blank which has overlap with the IS peak, but when the overlap was integrated, it was always less than 5% of the IS area (Fig. 1). Furthermore, no carry over was observed after injection of a solvent sample after the highest calibrator (Fig. 2).

#### Linearity and calibration

The calibration curves for the three replicates of accuracy and precision assessments in human plasma and the cross validation in rat plasma are presented in the supplement (S4, S5). For each individual run, no more than one sample was excluded based on the criterion that its deviation from the nominal concentration exceeded 15%, with a threshold of 20% at the LLOQ. All calibration curves consistently demonstrated a coefficient of determination (R^2^) of at least 0.99, indicating excellent linearity and reliability of the assay.

#### Accuracy and precision

The full three accuracy and precision runs in human plasma showed good accuracy and precision in all runs. All samples deviated by no more than 15% from the nominal concentration, or 20% at the LLOQ. Additionally, the %CV was below 15% for all quality control levels across each validation day and for interday accuracy. This was also the case for the cross validation run performed in rat plasma. The complete results are presented in Table 1.

**Table 1:** The results of the accuracy and precision runs for human plasma, along with the cross-validation conducted in rat plasma, are presented

Quality control level	Nominal concentration [mg/L]	Calculated concentration [mg/L]	Accuracy bias [%]	Precision [%CV]
Human plasma				
Day 1				
LLOQ	0.25	0.24	− 4.0	7.0
Low	0.75	0.72	− 3.5	1.7
Middle	7.5	7.64	1.9	4.4
High	12.75	11.94	6.4	3.3
Day 2				
LLOQ	0.25	0.29	16.0	6.2
Low	0.75	0.80	7.5	6.6
Middle	7.5	8.11	8.2	6.8
High	12.75	13.89	9.0	6.2
Day 3				
LLOQ	0.25	0.22	− 10.4	6.1
Low	0.75	0.71	− 5.6	3.9
Middle	7.5	7.51	0.1	3.9
High	12.75	13.02	2.1	2.7
All days				
LLOQ	0.25	0.25	0.5	12.9
Low	0.75	0.75	− 0.5	7.5
Middle	7.5	7.75	3.4	6.2
High	12.75	12.95	1.6	7.6
Rat plasma				
LLOQ	0.25	0.24	− 4.4	4.3
Low	0.75	0.75	− 0.1	2.7
Middle	7.5	7.64	1.9	3.4
High	12.75	13.41	5.2	2.7

For human plasma, three validation runs were completed on separate days. The data illustrate the intraday accuracy and precision for each validation day, as well as the interday accuracy and precision across all three runs. For the rat plasma, the results of a single run are shown. For all runs 5 replicates were measured per concentration level

#### Matrix and anticoagulant effect

No significant matrix or anticoagulant effects were observed, as indicated by both values being close to one, suggesting minimal interference with the detection of FSM. The variability among different individuals was also minimal, with a coefficient of variation (%CV) of 2.3% across all samples with a mean of the IS normalized matrix effect of 1.0. The same was true for the anticoagulant effect, which was 1.03 with 2.2% CV.

#### Stability

The observed samples both in human and rat plasma showed high stability (Table 2). No significant degradation was observed in any of the tested conditions, except the autosampler stability. In the autosampler degradation of above 15% was observed after 12 h. This is most likely due to the acidic conditions (low pH) created by the precipitation agent TCA. The degradation was mainly observed in FOF, explaining the apparent increase in concentration. Therefore, run times of analytical runs should be kept as short as possible, with a maximum of 10 h.

**Table 2:** Results of the stability test in human and rat plasma, respectively

			Human plasma		Rat plasma	
		Concentration	Accuracy bias	Precision	Accuracy bias	Precision
		µg/mL	%	%CV	%	%CV
Environment	Quality control level					
Autosampler 8 h (4 °C)	Low	0.75	1.8	2.2	–	–
	High	12.75	2.9	3.4	–	–
Autosampler 10 h (4 °C)	Low	0.75	7.1	5.6	–	–
	High	12.75	7.6	3.6	–	–
Autosampler 12 h (4 °C)	Low	0.75	17.3	4.9	–	–
	High	12.75	14.4	3.6	–	–
Bench-top (23 °C)	Low	0.75	− 0.2	4.7	0.0	4.7
	Middle	7.5	3.1	4.5	0.2	6.5
	High	12.75	1.5	2.6	− 4.2	3.8
Refrigerator (4 °C)	Low	0.75	− 4.7	4.0	− 0.5	5.6
	Middle	7.5	− 0.1	1.0	1.1	5.1
	High	0.75	− 0.5	1.7	− 2.3	C
Freeze–Thaw (− 80 °C)	Low	7.5	− 0.6	5.1	− 1.8	5.0
	Middle	0.75	1.4	4.1	0.7	5.0
	High	7.5	0.9	2.0	− 3.0	5.8

#### Accelerated long-term stability

**Fig. 3:**
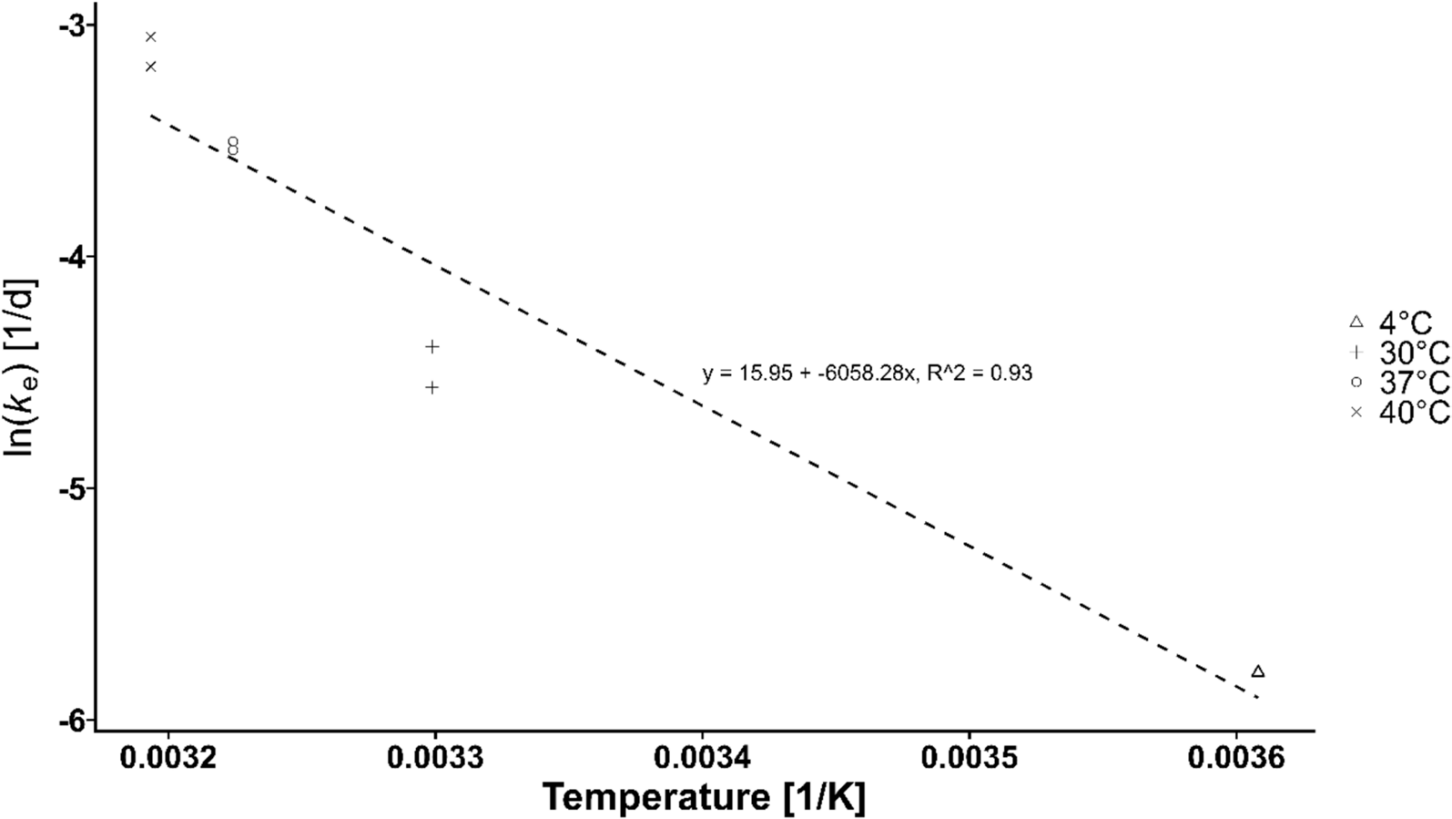
Arrhenius-Plot plotting the ln(k_e_) over T^−1^. Linear regression was performed to estimate the linear relationship between ln(k_e_) and T^−1^

The degradation of FSM could be adequately described with the assumption of a first-order process and showed equal degradation constants at both concentration levels. Complete results of the stability study are present in the supplement (S6, S7). The linear regression also showed a good fit in the Arrhenius-plot, with an R^2^ -value of 0.93 (Fig. 3). Using the linear equation of the regression, *ke* was calculated at − 20 °C and − 80 °C. When using the calculated *ke* to estimate the degradation, FSM was predicted to be stable at − 20 °C for up to one year, with an estimated degradation of approximately 12%. At − 80 °C, a degradation close to 0% was estimated even after 3 years. Calculated stabilities at different conditions and times are presented in the supplement (S8).

### Pharmacokinetic applications

#### Pharmacokinetic application in humans

**Fig. 4:**
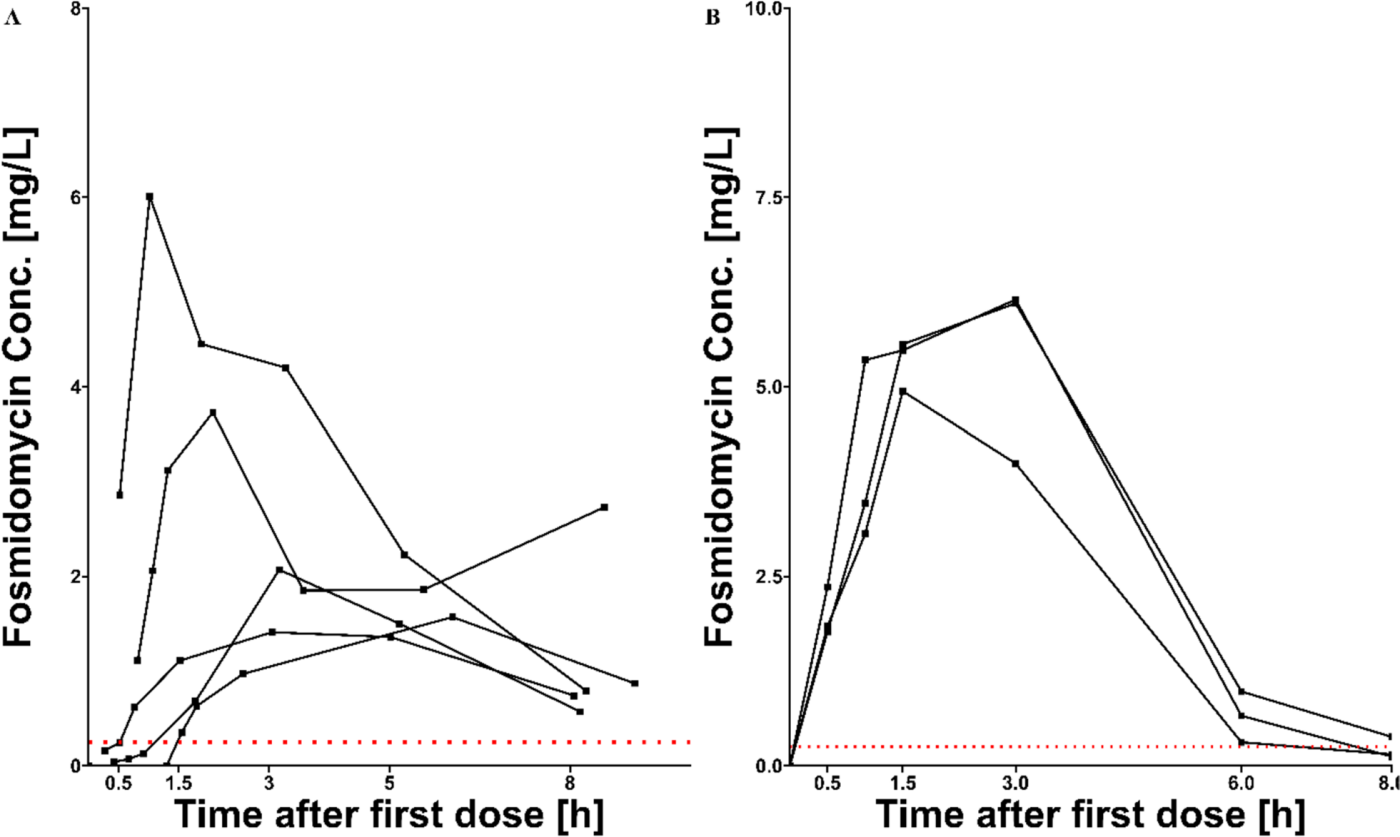
**A** Concentration–time profiles of 5 adults receiving between 1575 and 2550 mg of FSM (30 mg/kg). **B** Concentration–time profile of three male Wistar rats receiving a 5 mg dose of fosmidomycin. In both plots the black squares represent the measured samples and the red doted line shows the LLOQ

The pharmacokinetic concentration time data of 5 adults receiving doses between 1575 and 2550 mg of FSM (30 mg per kg) is shown in Fig. 4. The results of the PK analysis are shown in Table 3.

**Table 3:** Results of the PK analysis of the human and rat study. Results are presented as geometric mean (geometric standard deviation)

PK study in humans	
C_max_ [mg/L]	2.53 (1.85)
AUC_0-8 h_ [mg/L∙h]	12.3 (1.62)
T_max_ [h]	2.20 (2.09)
T_1/2_ [h]	3.16 (1.48)
PK study in rats	
C_max_ [mg/L]	5.70 (1.13)
AUC_0-8 h_ [mg/L∙h]	19.5 (1.25)
T_max_ [h]	2.38 (1.50)
T_1/2_ [h]	1.09 (1.20)

#### Pharmacokinetic application in rats

The pharmacokinetic profiles of three Wistar rats were assessed following the oral administration of 15 mg of FSM, with the concentration–time data illustrated in Fig. 4. The results of the PK analysis are shown in Table 3.

## Discussion

The present bioanalytical assay for FSM was successfully developed and validated in accordance with the EMA guidelines for bioanalytical method validation [11]. The assay is characterized by its speed, robustness, and sensitivity, making it well-suited for the pharmacokinetic analysis of FSM. Moreover, the assay demonstrated its flexibility by being effective across multiple matrices. The assay was successfully applied in two pharmacokinetic studies: one involving humans with uncomplicated malaria and the other using healthy Wistar rats.

The method offers an improvement in the limit of quantification, outperforming the published disk diffusion assay by a factor of four [9] and capillary electrophoresis assays by a factor of two [10], therefore enabling more precise measurements of the FSM absorption and elimination phase in pharmacokinetic studies. Moreover, sample preparation is considerably quicker compared to capillary electrophoresis and exhibits enhanced selectivity compared to UV detection. The disk diffusion assay avoids the step of sample preparation, but requires a labour-intensive setup of the assay. The disk diffusion assay is not suitable for samples containing other antibiotics to which the test organism is sensitive. Our LC–MS-based approach improves preparation efficiency and robustness, and allows for broader applicability in complex matrices and the co-administration of other drugs.

The method involves a straightforward extraction process using a strong acid to precipitate proteins from plasma. Organic solvents were explored, but resulted in inferior retention, sensitivity, and high variability when the aqueous content in samples fluctuated, due to FSM's poor solubility in organic solvents. A significant drawback of the selected method is the harsh acidic environment (low pH) in the final sample, which leads to stability issues. This necessitates constant cooling of the prepared sample and limits the possible runtime to 10 h. Although alternative extraction techniques, such as solid-phase extraction, may enhance stability and sensitivity, they come at a higher cost and necessitate a more time-consuming sample preparation.

The development of the assay presented significant challenges due to the physicochemical properties of FSM, which complicated the creation of a reliable LC–MS method. As a highly polar compound, FSM was evaluated with HILIC columns (specifically amide and Z-type columns), but neither yielded clean, distinct peaks. Additionally, a mixed-mode column with anion exchange properties also failed to provide satisfactory results. A comprehensive evaluation was conducted using various buffer concentrations (ammonium formate), different pH values, and multiple gradient configurations. Ultimately, only the use of 100% aqueous buffer in the selected column produced consistent retention and high-quality peaks. Furthermore, optimization of the mass spectrometry parameters through automatic flow injection analysis enhanced the sensitivity of the method, resulting in the determination of the final LLOQ.

Stability assessments revealed that FSM displays excellent stability under various storage conditions. The accelerated long-term study predicts prolonged storage at − 80 °C, which indicates negligible degradation even after 3 years. This finding is crucial for ensuring the integrity of pharmacokinetic samples stored for extended periods, particularly in multicentric trials involving international transport. Bronner et al. also investigated the long-term stability of FSM (in human serum) and found some degradation at − 80 °C after 3 months ranging from -5.5 and − 14.6% using an capillary electrophoresis–UV assay [10]. However, the stability measurements shown in the study showed high variability between the measured concentration even showing an increase in concentration in one case. This suggests that there may be additional processed involved than sole degradation of FSM and other components might affect the UV detection used in their study.

The pharmacokinetic application of the method produced viable concentration–time profiles for FSM. In comparison to a prior study conducted on adults with acute uncomplicated malaria in Thailand, where participants received a fixed dose of 1200 mg FSM (corresponding to a mean dose of 22 mg/kg), the presented results demonstrate lower values for both Cmax and AUC_0-8 h_, even at the higher administered dose of 30 mg/kg. The Thai study reported a Cmax of 4.58 mg/L (95% CI 2.32–6.77) and an AUC_0-8 h_ of 22.91 mg/L∙h (95% CI 12.65–33.32) [14]. In this study, the mean values for AUC_0-8 h_ and Cmax were significantly lower with 12.3 mg/L h and 2.53 mg/L respectively. However, it is noteworthy that two of the five patients demonstrated values that were relatively close to those reported in the Thai study, while three patients exhibited markedly lower values. One possible explanation for these discrepancies may stem from the sampling scheme, which could have missed capturing the Cmax. In the Thai study, the peak concentration was detected at 2 h post-administration. In contrast the sampling scheme in the presented study sampled at 1.5- and 3 h. Furthermore, significant differences in the study populations may influence the results, as this study was conducted in Gabon. The method employed to quantify the samples may also contribute to the observed differences. Specifically, if the disk diffusion assay used has an inherent bias, this could further explain the variability in the results. Notably, it was observed that the estimated half-life of the drug in the present study was comparable to that reported in the Thai study, measuring 3.4 h aligning with their value of 3.1 h.

The pharmacokinetic findings for FSM in rats reveal Cmax values that are lower than those reported in a previous study involving 6-week-old male JC1:SD rats receiving a dose of 100 mg/kg. In that study, peak serum concentrations were found to be approximately 8 to 9 mg/L between 1 and 2 h post-administration [15]. However, these values are not directly comparable, as the exact dosage administered in our study remains unknown. While the half-life in rats was not established in the prior study, reported half-lives for dogs and humans were 1.99 h and 1.87 h, respectively. Notably, these half-lives are lower than the one observed in the human study. The estimation of the half-life in the rat study should be interpreted with caution due to the limited data available during the elimination phase.

Despite some samples being below the limit of quantification, most of FSM's absorption and elimination phases were captured. Given the limited number of samples within the range of 0.25 to 0.5 mg/L, the improved limit of quantification was not utilized in this analysis. However, with the enhanced understanding of FSM's pharmacokinetics, the sampling scheme can be refined in future pharmacokinetic studies to capture more data in this area. The findings demonstrate that the assay can be used in studies to further understand the pharmacokinetics of FSM. Furthermore, the assay can be used for samples in rat plasma, allowing for use in preclinical development, for example for exploring new drug formulations.

The developed LC–MS/MS method represents a significant advancement in the bioanalytical quantification of FSM, and it is promising to enhance the pharmacological understanding and development of FSM as a feasible antimalarial agent. This research establishes a foundation for extended pharmacokinetic modeling, dose optimization, and combination therapeutic strategies against malaria. Future research directions may include extending this method to other biological fluids and optimizing the sample preparation to allow for even more sensitivity and a more stable final sample.

## Conclusion

A robust assay for the quantification of FSM was developed which is applicable in multiple matrices and is thus applicable at multiple stages of pre-clinical and clinical development of the drug. As FSM is a drug which is yet to be developed in to a licensed drug studies on the pharmacokinetics of the drugs are essential, and this assay allows for accurate and precise measurements of FSM concentrations.

## Supplementary Information

Supplementary material 1.

## Data Availability

The data that support the findings of this study are available from the corresponding author, [SW], upon reasonable request.

## Abbreviations

LLOQ: Lower limit of quantification
LC–MS/MS: Liquid chromatography- tandem mass spectrometry
FSM: Fosmidomycin
IS: Internal standard
FOF: Fosfomycin
TCA: Trichloroacetic acid
EMA: European Medicines Agency
CV: Coefficient of variation
AUC_0-8__ h_: Area under the curve from 0 to 8 h
Cmax: Maximal concentration
