# Supplementary material for: Development and application of an LC–MS/MS method for quantification of fosmidomycin in human and rat plasma
Source: Malar J. 2025 Jul 25;24:243. doi: 10.1186/s12936-025-05489-1 (PMC12297723; doi:10.1186/s12936-025-05489-1)
Supplement: Supplementary file 1 — Supplementary material 1. [file 12936_2025_5489_MOESM1_ESM.docx]

**S1** Source parameters of the method.

| Curtain Gas (psi) | Collision Gas | Ion Spray Voltage (volt) | Temperature (°C) | Nebulizer Gas (psi) | Heater Gas (psi) |
| --- | --- | --- | --- | --- | --- |
| 20 | Low | -4500 | 500 | 60 | 80 |

**S2** Fragments and parameters used for each analyte and the internal standard (IS)

| Analyte | Q1 Mass (Da) | Q3 Mass (Da) | Dwell Time (msec) | Declustering Potential (volts) | Entrance Potential (volts) | Collision Energy (volts) | Collision Cell Exit Potential (volts) |
| --- | --- | --- | --- | --- | --- | --- | --- |
| Fosmidomycin quantifier | 181.913 | 135.900 | 150 | -100 | -10 | -18 | -13 |
| Fosmidomycin qualifier | 181.913 | 78.8 |  |  |  |  |  |
| Fosfomycin (IS) | 136.884 | 78.900 | 150 | -100 | -10 | -38 | -11 |

**S3** Temperatures are shown with their respective sampling times. At each sampling time triplicates of LQC and HQC were measured.

| **Condition** | **Baseline** | **Sample 1** | **Sample 2** | **Sample 3** | **Sample 4** |
| --- | --- | --- | --- | --- | --- |
| **temperature** | **time** | **time** | **time** | **time** | **time** |
| **°C** | **days** | **days** | **days** | **days** | **days** |
| 4 | 0 | 15 | 35 | 56 | - |
| 23 | 0 | 7 | 15 | 21 | 50 |
| 30 | 0 | 8 | 14 | 21 | 28 |
| 37 | 0 | 7 | 15 | 21 | 50 |
| 40 | 0 | 8 | 14 | 21 | 28 |


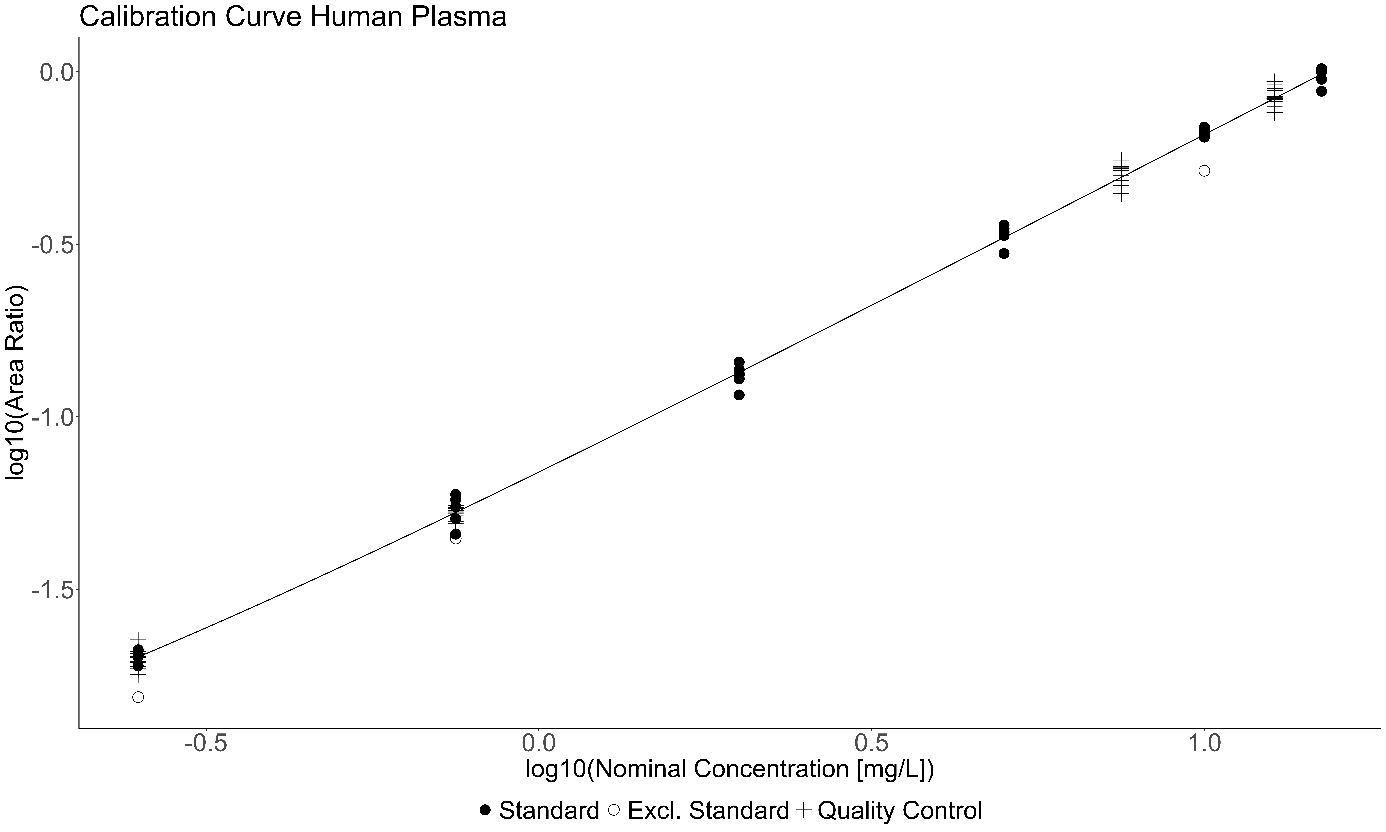


**S4** Calibration of fosmidomycin in human plasma: y=0.00379*x + 0.00379 (r=0.99734, r2=0.99468) (weighting: 1/x2). The calibration curve is fitted over all three validation days and shows the quality control samples of the three validation days. The calibration curve is shown in log10-log10 transformation for easier readability.

**
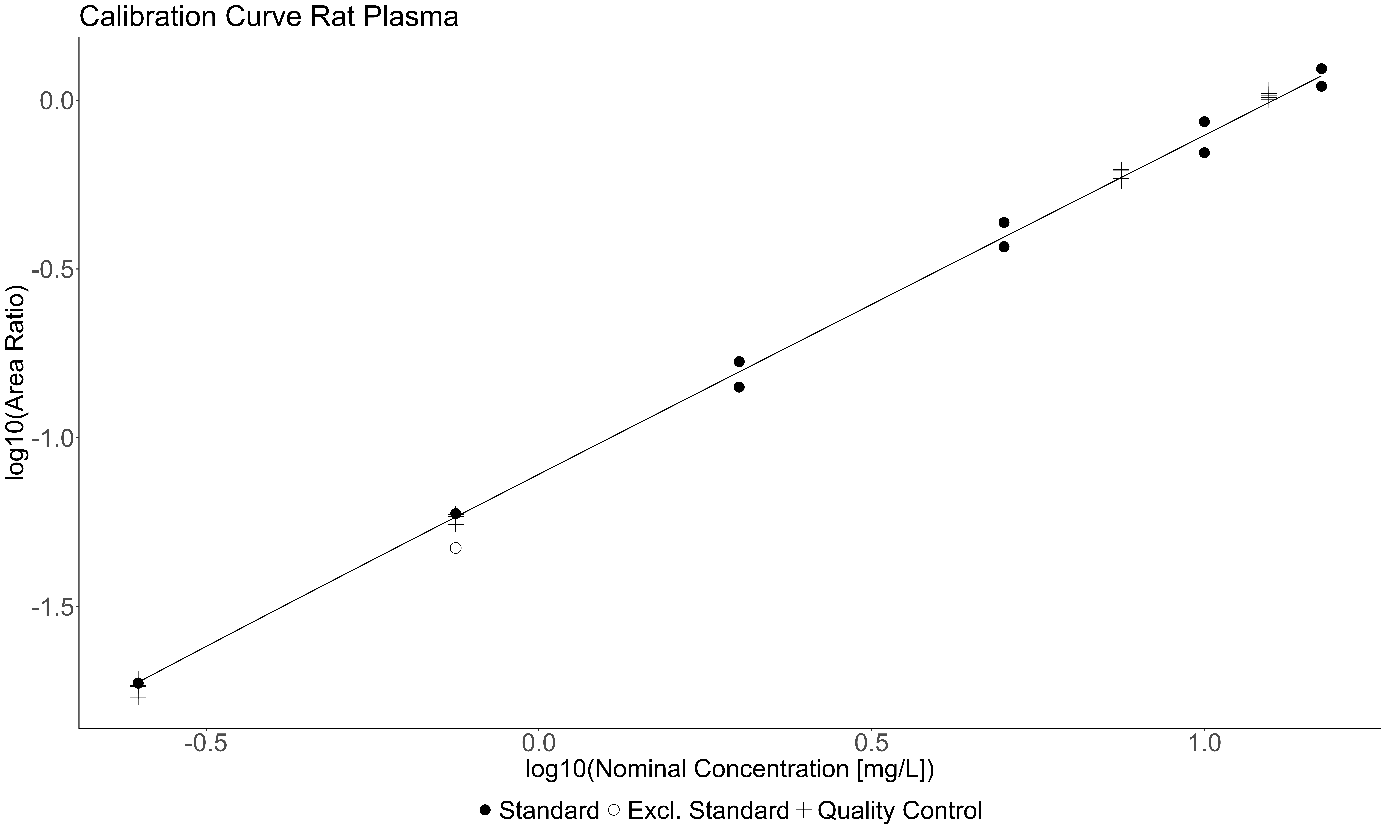
**

**S5** Calibration of fosmidomycin in rat plasma: y=0.0.07878*x – 8.362e^-4^ (r=0.99594, r^2^=0.99190) (weighting: 1/x^2^). The calibration curve is shown in log10-log10 transformation for easier readability.

**S6** Results of the degradation measurements. All samples unless otherwise noted were measured in triplicates.

|  |  | **Baseline** | **Sample 1** | **Sample 2** | **Sample 3** | **Sample 4** |  |
| --- | --- | --- | --- | --- | --- | --- | --- |
| **Variable** |  | **Concentration** | **Concentration** | **Concentration** | **Concentration** | **Concentration** |  |
| **Unit** |  | **µg/mL** | **µg/mL** | **µg/mL** | **µg/mL** | **µg/mL** |  |
| **Storage** |  |  |  |  |  |  | **Ke** |
| Refrigerator  (4°C) | **time** | **0** | **15** | **35** | **56** | **-** |  |
|  | LQC | 0.71 | 0.7 | 0.64 | 0.60 | - | -0.003 |
|  | HQC | 12.22 | 11.74 | 10.90 | 10.34 | - | -0.003 |
| Incubator  (37°C) | **time** | **0** | **7** | **15** | **21** | **50** |  |
|  | LQC | 0.72 | 0.59 | 0.47 | 0.39 | 0.17 (n=1) | -0.029 |
|  | HQC | 12.47 | 9.61 | 7.86 | 6.76 | 2.66 | -0.030 |
| Drying cabinet  (30°C) | **time** | **0** | **8** | **21** | **28** | **-** |  |
|  | LQC | 0.74 | 0.69 | 0.58 | 0.56 | - | -0.010 |
|  | HQC | 11.99 | 11.32 | 9.26 | 8.62 | - | -0.012 |
| Drying cabinet  (40°C) | **time** | **0** | **8** | **21** | **28** | **-** |  |
|  | LQC | 0.81 | 0.59 | 0.3 | 0.22 | - | -0.047 |
|  | HQC | 12.30 | 9.48 | 5.08 | 3.98 | - | -0.042 |


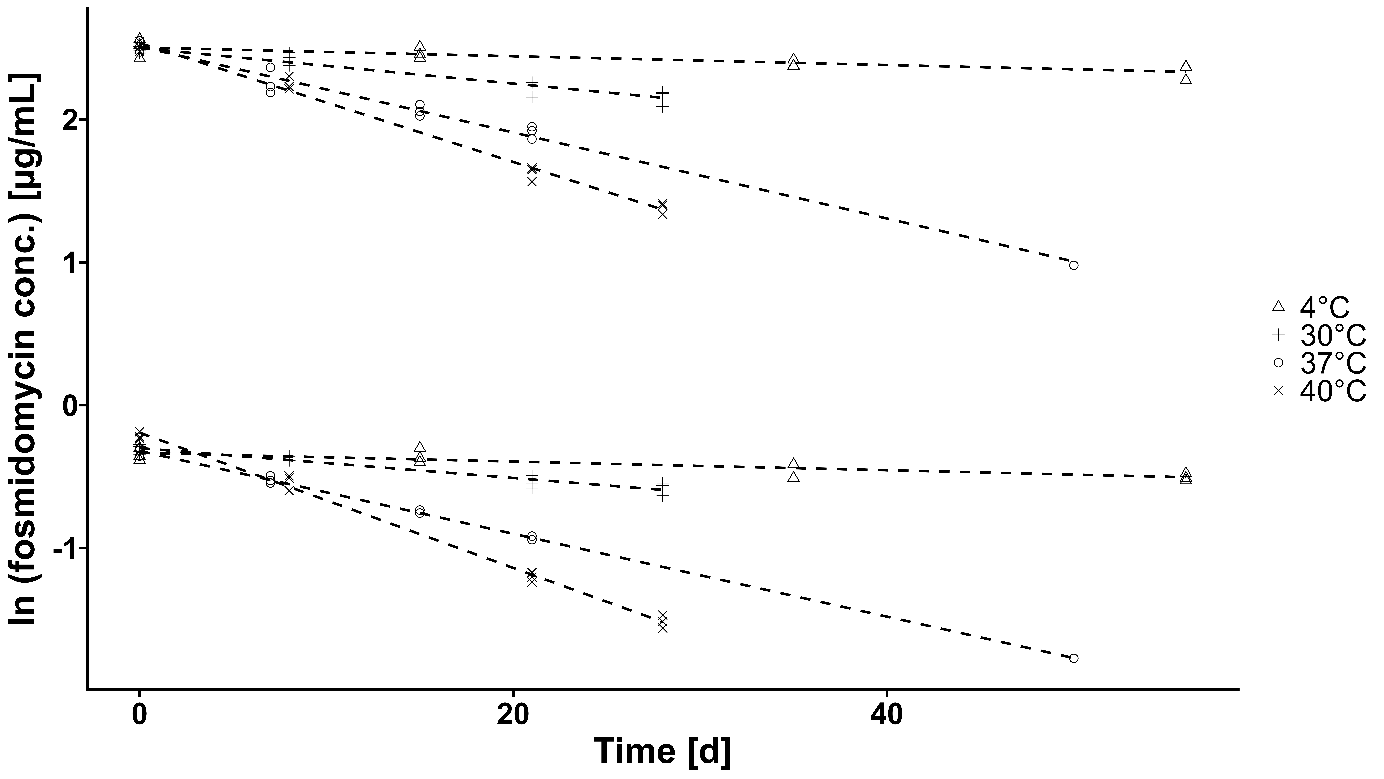


**S7** Plots showing degradation of fosmidomycin at different temperatures and concentrations. The slope represents the degradation constant k_e_ and is shown in S6.

**S8** Extrapolation of long-term fosmidomycin stability in human plasma. k_e_ for both temperatures are calculated from the Arrhenius-plot.

| **Variable** | ***k*_e_** | **Time** | **Level** | **Initial Concentration** | **Calculated Concentration** | **Degradation** |
| --- | --- | --- | --- | --- | --- | --- |
| **Unit** | **[1/d]** | **d** |  | **µg/mL** | **µg/mL** | **%** |
| **Environment** |  |  |  |  |  |  |
| Freezer  (-20°C) | 0.000343139 | 365 | LQC | 0.75 | 0.66 | 12.0 |
|  |  |  | HQC | 12.75 | 11.25 | 11.8 |
|  |  | 730 | LQC | 0.75 | 0.58 | 22.7 |
|  |  |  | HQC | 12.75 | 9.92 | 22.2 |
|  |  | 1095 | LQC | 0.75 | 0.52 | 30.7 |
|  |  |  | HQC | 12.75 | 8.76 | 31.3 |
| Freezer  (-80°C) | 2,02713e-07 | 365 | LQC | 0.75 | 0.75 | 0 |
|  |  |  | HQC | 12.75 | 12.75 | 0 |
|  |  | 730 | LQC | 0.75 | 0.75 | 0 |
|  |  |  | HQC | 12.75 | 12.75 | 0 |
|  |  | 1095 | LQC | 0.75 | 0.75 | 0 |
|  |  |  | HQC | 12.75 | 12.75 | 0 |
